# Wear Performance of Calcium Carbonate-Containing Knee Spacers

**DOI:** 10.3390/ma10070805

**Published:** 2017-07-15

**Authors:** Ulrike Mueller, Joern Reinders, Sydney Smith-Romanski, Jan Philippe Kretzer

**Affiliations:** Laboratory of Biomechanics and Implant Research, Clinic for Orthopedics and Trauma Surgery, Heidelberg University Hospital, Schlierbacher Landstrasse 200a, Heidelberg 69118, Germany; ulrike1.mueller@med.uni-heidelberg.de (U.M.); joern-reinders@gmx.de (J.R.); sydney.smith-romanski@uconn.edu (S.S.-R.)

**Keywords:** total knee arthroplasty, bone cement, spacer, wear, particles

## Abstract

Articulating spacers should be wear-resistant and load-bearing to avoid prolonged immobilization of the patient and to reduce morbidity. However, due to the articulation of both components, a release of cement wear particles is to be expected. The aim of this study was to investigate the wear performance of a new spacer cement that contains calcium carbonate as a radio-opaque substance, in comparison to an established barium sulphate-containing spacer material, and also to characterize the amount, morphology, and size distributions of the released cement particles in detail. Force-controlled simulation was carried out on an AMTI knee simulator. The test parameters were in accordance with the standard ISO 14243-1 with a 50% reduced axial force. Tests were run for 500,000 cycles at a frequency of 1 Hz. For wear analysis, photographic documentation of the wear scars, gravimetric wear measurements and wear particle analysis were performed. The barium sulphate spacer material showed a total articular wear of 375.53 ± 161.22 mg. For the calcium carbonate-containing cement, reduced articular wear of 136.32 ± 37.58 mg was determined. Isolated cement wear particles of the barium sulphate-containing cement had a diameter of 0.429 ± 0.224 μm and were significantly larger compared to the calcium carbonate-containing cement (0.380 ± 0.216 μm, *p* = 0.02). The calcium carbonate-containing cement showed better wear performance in terms of gravimetric wear and particle release. Thus, calcium carbonate seems to be a promising material as a radio-opaque substrate in cement spacers.

## 1. Introduction

Periprosthetic infection is a serious and devastating complication in total hip and knee joint replacements, and one of the major reasons for revision surgery in joint arthroplasty [1,2].

The preferred treatment option for chronic infection of a joint replacement is the use of antibiotic-impregnated polymethylmethacrylate (PMMA) bone cement spacers in a two-stage re-implantation procedure [3,4,5].

Spacers may be articulating or non-articulating. Which type of spacer is used depends on several factors, including the condition of the soft tissues, the amount of bone loss, or the need for joint motion [6]. Advantages of articulating mobile spacers include the possibility for the patient to move during the interim period, which may decrease the risk of muscle contracture due to immobilization and improve postoperative range of motion [3]. However, due to the articulation of both cement components, a release of cement wear particles is expected [7]. These particles may have several biological consequences such as an involvement in proinflammatory reactions or osteolysis [8,9]. Moreover, cement particles might also be involved in mechanical degradation processes, as the cement typically contains radio-opaque substances such as barium sulphate or zirconium dioxide [10]. Cement and zirconium dioxide particles from spacers have been detected in the synovial membrane when the spacers are removed for the second stage of the revision [7]. Therefore, it is expected that cement particles may also contribute to the wear performance of the final joint replacement. As third-body wear particles, they are able to accelerate wear processes, particularly when radio-opaque substances such as barium sulphate or zirconium dioxide are involved [11,12].

So far, little is known about the wear performance of articulating spacer materials [13,14,15]. In particular, detailed information regarding the particle size, particle morphology, and amount of abraded particles are missing, which are important parameters regarding the potential for proinflammatory reactions [16]. Moreover, a new cement material has been developed that contains hydrophilic and soft calcium carbonate as a radio-opaque substance instead of the established hard substances. Therefore, the aim of this study was to investigate the wear performance of this new material in comparison to an established barium sulphate-containing spacer cement and to characterize the amount, morphology, and size distributions of the released cement particles in detail.

## 2. Material and Methods

### 2.1. Test Specimens

**Figure 1:**
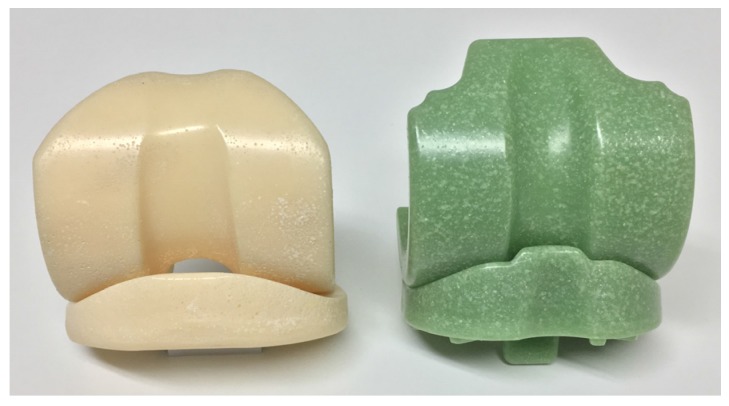
Articulating components of the two cement spacers: BS-cement (left) and CC-cement (right).

The calcium carbonate-containing material (CC-cement) was developed by Heraeus Medical (Wehrheim, Germany) and the commercially available barium sulphate-containing (BS-cement) Spacer K from TECRES (Verona, Italy) was used as a control material. Both articulating spacers were ready-made for clinical application (so-called “preformed”) and consist of a medium-sized tibial and femoral component of similar dimensions (Figure 1). Further characteristics of the analyzed material are given in Table 1.

The initial cement topography was evaluated using a tactile roughness measurement instrument (Perthometer M2; Mahr, Göttingen, Germany; accuracy: 12 nm). The results are given in Table 2.

The roughness values were comparable between both cements, except the Rz values, which are slightly higher for the BS-cement (significant difference only for Rz of the condyle (*p* = 0.005)).

### 2.2. Wear Simulation

**Figure 2:**
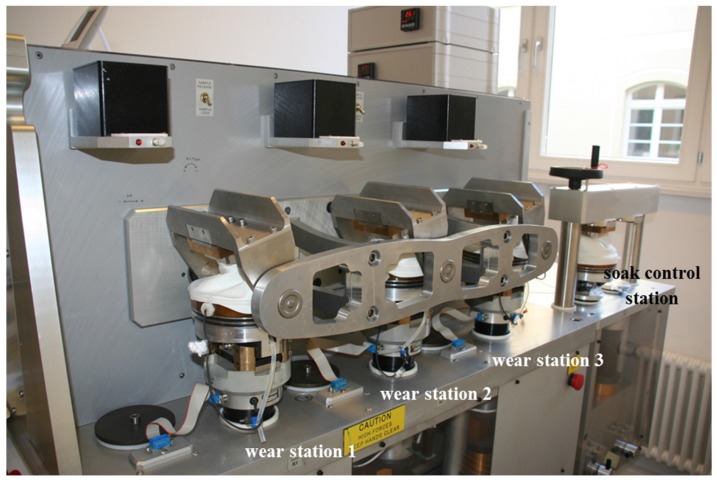
Knee wear simulator with three wear stations and one soak control station.

To simulate knee motion and loadings, a wear simulator (model KS2-6-1000, Advanced Mechanical Technology Inc., Watertown, MA, USA) was used (Figure 2). In addition to the three wear stations, a reference station was available for soak control, which was only axially loaded. In each group, three spacer systems were used for wear evaluation and one additional spacer served as a soak control to account for liquid absorption of the cement. The wear tests were performed in force control according to ISO 14243-1:2009, with a 50% reduced axial force to consider the partial weight bearing of the patient. The simulations were carried out at 37 ± 1 °C in closed chambers filled with calf serum (PAA Laboratories GmbH, Pasching, Austria). The serum had a protein concentration of 20 g/L [17], and 1.85 g/L of sodium azide (NaN3) and 7.44 g/L of ethylene diamine tetraacetic acid (EDTA) were added to the calf serum to retard bacterial growth and to prevent the formation of calcium phosphate films on the surface. Tests were run at a frequency of 1 Hz for a total of 500,000 cycles. This number of loading cycles was chosen because it represents a duration of three to six months of clinical use [18].

### 2.3. Wear Analysis

For wear analysis, photographic documentation of the wear scars, gravimetric wear measurements according to ISO 14243-2, and wear particle analysis according to ASTM F1877-05 were performed. 

#### 2.3.1. Photographic Documentation

After test termination, the worn surfaces were photographically documented using a digital camera (EOS 1100D, Canon Inc., Tokyo, Japan).

#### 2.3.2. Gravimetric Wear Measurements

Prior to wear testing, all samples were pre-soaked in calf serum until saturation in weight increase was observed. Gravimetric wear measurements were performed after test termination at 500,000 cycles. First, the specimens were repeatedly cleaned in an ultrasonic bath. Next, the samples were dried in a vacuum drying chamber for 30 min before they were weighed using a high precision balance with a measuring accuracy of 15 μg (Sartorius Genius ME235S-OCE, Sartorius AG, Göttingen, Germany). Measurements were performed in a temperature-controlled measuring room (21 ± 1 °C). Every specimen was weighed five times in rotation with the other specimens. During the weighing procedure, the spacers were rinsed with nitrogen to prevent electrostatic charging. Wear was determined by adding the weight gain of the soak control to the weight loss of the test specimens in order to account for liquid absorption.

#### 2.3.3. Particle Analysis

Wear particles were isolated from the serum lubricants after test termination. The samples were diluted with ultrapure water, following a digestion with 37% hydrochloric acid. The samples were heated to 60 °C and stirred with a magnetic stir bar rotating at 400 rpm. for 60 min. A sample of 0.3 mL was filtered with methyl alcohol onto an alumina filter with a pore size of 0.1 μm.

Particles were analyzed using high resolution, field emission gun scanning electron microscopy (FEG-SEM) (Leo 1530, Leo, Oberkochen, Germany), and digital image processing software (Leica Qwin V3, Leica Microsystems, Wetzlar, Germany). A magnification of 10,000× was used. For both spacer cements, three filters were analyzed and three randomized images were taken on each filter to obtain a representative particle size distribution. To describe the size and morphology of the particles, the equivalent circle diameter (ECD), aspect ratio (AR), form factor (FF) and roundness (R) were determined according to ASTM F 1877-98. Furthermore, the estimated total number of particles was calculated based on the number of particles recovered on the filter, the analyzed area, the dilution, and the total serum volume.

### 2.4. Statisitical Analysis

All data are presented as means ± standard error of the mean (SEM). Error bars indicate the standard error of mean. The normal distribution of data was confirmed using the Kolmogorov-Smirnov test. To compare gravimetric wear as well as the morphological characteristics of the particles, a student’s *t*-test for two independent parametric samples was calculated. All tests were two-sided and performed at a significance level of *p* ≤ 0.05. Statistical analysis was performed with SPSS (version 23, SPSS Inc., Chicago, IL, USA).

## 3. Results

### 3.1. Photographic Documentation

**Figure 3:**
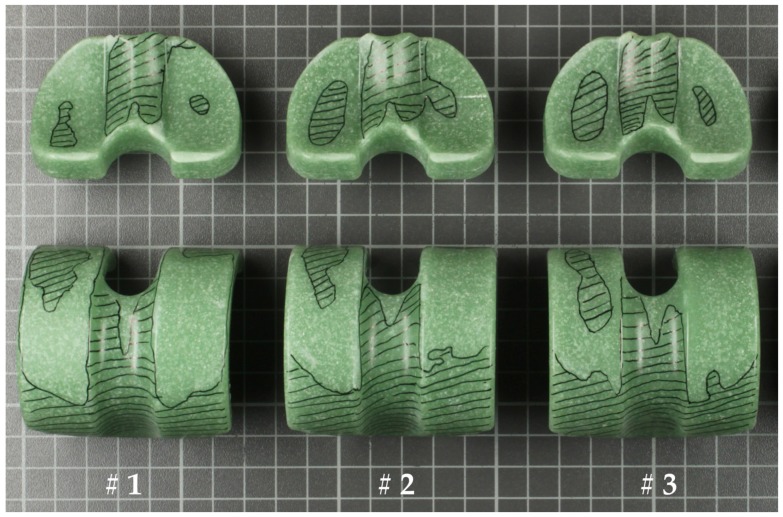
Worn areas of the three (#1–#3) tested CC-cement specimens.

**Figure 4:**
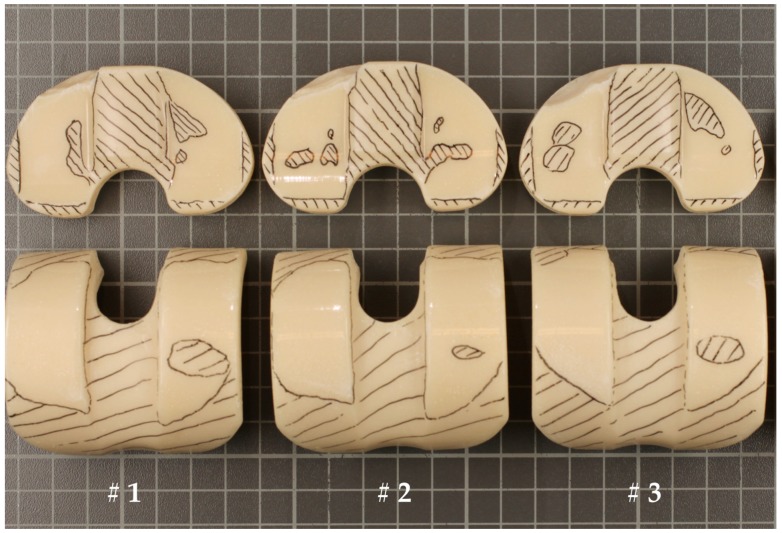
Worn areas on the three (#1–#3) tested BS-cement specimens.

Figure 3 and Figure 4 show a global view of the worn areas of the both cement spacers after 500,000 cycles. The non-worn areas were marked black (hatched). Subjectively, the wear scars on the medial side of the components are more severe, and this applies to both cements.

### 3.2. Gravimetric Measurements

**Figure 5:**
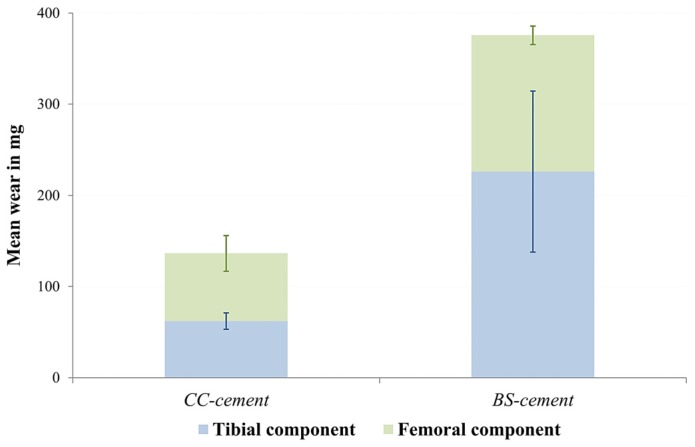
Comparison of the wear results of the tested femoral and tibial components of the two different cements. Error bars represent the standard error of mean.

The results of the gravimetric wear measurements are shown in Figure 5. The CC-cement showed a total wear reduction of 64% compared to the BS-cement. Regarding the femoral components, the CC-cement wear was measured at 74.3 (±19.5) mg compared to 149.6 (±10.0) mg for the BS-cement. This difference was statistically significant (*p* = 0.027). For the tibial components, wear was measured at 62.0 (±9.1) mg for the CC-cement and 226.0 (±88.3) mg for the BS-cement (*p* = 0.203).

### 3.3. Particle Analysis

**Figure 6:**
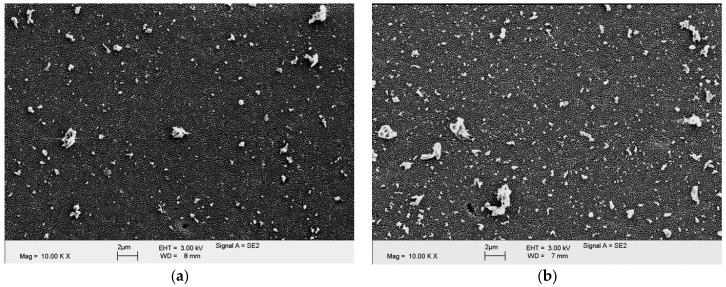
Isolated wear particles of the CC-cement (**a**) and the BS-cement (**b**).

**Figure 7:**
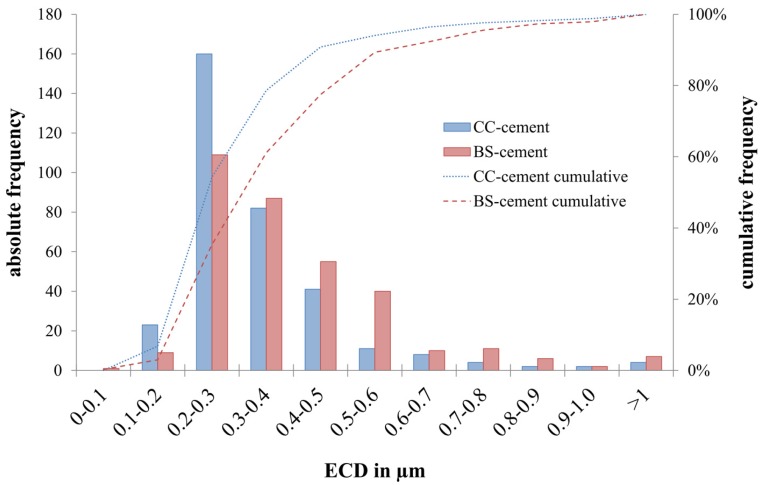
Mean equivalent circle diameter distribution of both cements.

Examples of the isolated and analyzed particles for both cements are shown in Figure 6. The particle characteristics are summarized in Table 3. For the BS-cement, the estimated total number of released particles was increased by 47% compared to that for the CC-cement. However, this difference was statistically insignificant. Significant larger particles were found for the BS-cement, which can also be seen in the particle size distribution (Figure 7). Regarding the particle shape parameters such as the aspect ratio, roundness, and form factor, no relevant differences were observed between both cements.

## 4. Discussion

**Figure 8:**
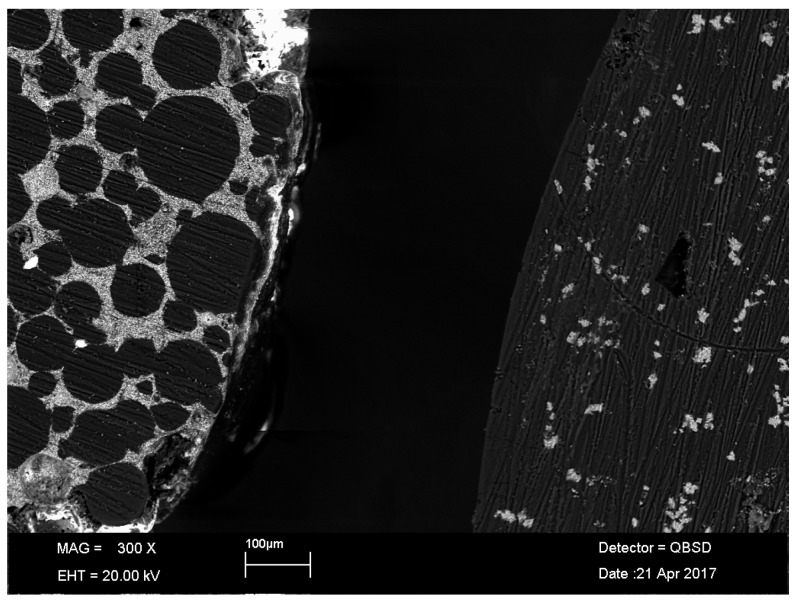
Sections of both cements analyzed in an SEM. Left: BS-cement, Right: CC-cement.

This study confirms that the newly developed calcium carbonate-containing cement reduces the amount of wear and released particles. This behavior can be explained by the use of the soft calcium carbonate as a radio-opaque substance in the spacer cement, which seems to be less abrasive against the surfaces of the sliding partners compared to barium sulphate-containing spacer cements. Due to the calcium carbonate structure, intracrystalline shearing may be beneficial as the calcium carbonate particles may be grinded when they are released from the surface. The current findings might also be explained by differences in the microstructure of both cements (Figure 8). For the CC-cement, a fine dispersion of the calcium carbonate within the PMMA was confirmed by EDX analysis. For the BS-cement, unpolymerized PMMA beads were observed within a barium sulphate-containing matrix. It seems that the PMMA swelling, which occurs during the polymerization process, has not affected all portions of the PMMA powder. It can be assumed that these PMMA beads detach more easily from the bulk material during articulation, leading to the higher wear of the BS-cement in comparison to the CC-cement.

The current results are in good agreement with other studies, although very few studies on knee spacer wear are available. Regarding the effect of radio-opaque substances on the wear behavior of the knee spacer, only one study has been published. Bitsch et al. [13] compared the wear performance of a calcium carbonate to a zirconium dioxide-containing cement on a knee wear simulator. Although they did not determine neither the amount of wear nor the particle characteristics, they reported smaller wear scar areas on the knee spacer components for the calcium carbonate cement. Villa and Carnelli [15] evaluated the wear performance of a barium sulphate-containing material and reported a lower wear amount for the same type and size of spacer as in the current study. However, there are several differences in the applied method. For example, in the current study, internal-external torque and anterior-posterior force were additionally applied. Furthermore, Villa and Carnelli determined the wear amount by isolating and weighting the particles. Due to the applied particle centrifugation and the missing consideration of liquid absorption, the wear amount could be underestimated in their study. In addition, only one sample was analyzed for the corresponding size. Even though Affatato et al. [14] studied the wear performance of hip cement spacers, the findings regarding the wear amount of the same cement material are in accordance with the current results.

Although there was a remarkable wear reduction due to the calcium carbonate in this study, the wear amount of both studied materials was higher compared the polyethylene wear in conventional knee arthroplasty (Table 4).

Several studies have shown that cement particles may induce proinflammatory reactions and macrophage activation, potentially leading to osteolysis and bone resorption [25,26,27,28]. In this regard, higher particle concentrations increase the proinflammatory potential of the wear debris [25,28]. Furthermore, Mitchell et al. [28] studied the effect of the cement particle size on the primary human macrophage TNF-α production and determined that the particle size range between 0.1 and 1 μm is the most aggressive. Although there was a difference in cement particle size in the current study, both particles sizes are in the reported range of increased proinflammation [28]. Data on proinflammatory effects depending on the shape for cement particles are currently unavailable. However, if studies regarding the particle shape of other materials such as polyethylene are considered, rough and elongated particles have been identified to increase adverse tissue responses [29]. The currently analyzed cement particles are far from perfectly round, smooth particles, and are more similar in shape and morphology to polyethylene particles [23]. 

It is expected that abraded cement particles remain in the surrounding tissue. Substantial amounts of cement particles and zirconium dioxide were found in the synovial membrane six weeks after implantation when the spacers were removed for the second stage of the revision [7]. In this context, it must be considered that these particles may affect the final joint replacement. If cement particles enter the joint articulation, they may have an abrasive effect on the metallic femoral components, leading to increased surface roughness [30,31]. Consequently, the polyethylene wear may be significantly increased. Retrieval studies have shown a relation between third-body particles, increased roughness of the femoral condyles, and increased polyethylene wear [32,33]. This has also been confirmed by in vitro wear studies that determined increased polyethylene wear in the presence of cement particles in knee joint replacements [11,12].

The results of the current study are limited to the investigated materials and should not be generalized. Furthermore, as in every experimental setting, further limitations need to be considered. For example, gravimetric wear showed a large range in results that may have been related to the soaking behavior of the spacer material. A longer testing period would have likely reduced this effect. However, other studies were also limited regarding the testing duration and showed higher variations in the wear results [13,15]. Another aspect, which has not yet been taken into account, is the potential release of gentamicin that may have also influenced the gravimetric results. However, as the gentamicin concentration was low (2.5% *w*/*w*), this effect is not expected to have had a high impact. Besides the wear performance, further studies are essential in proving the mechanical stability and the antibiotic release kinetics for the calcium carbonate-containing spacer cement. Strengths of the study are that the particle release of articulating cement spacers was evaluated for the first time and that the gravimetric wear was analyzed separately for each component.

## 5. Conclusions

In conclusion, the new calcium carbonate-containing material showed better wear performance in terms of gravimetric wear and particle release. Thus, calcium carbonate seems to be a promising material as a radio-opaque substrate in cement spacers. Nevertheless, the released amount of cement particles is higher compared to conventional polyethylene for both investigated spacer materials. As a clinical consequence, excessive debridement during the removal of the cement spacer components is suggested in order to reduce the risk of third-body wear of the final joint replacement.

## Figures and Tables

**Table 1:** Characteristics of the two investigated spacer cements.

Characteristics	CC-Cement (New Material)	BS-Cement (Commercial Available)
Manufacturer	Heraeus Medical, Germany	Spacer K, TECRES, Italy
Cement	Polymethylmethacrylate (PMMA)	Polymethylmethacrylate (PMMA)
Radio-opaque substance	Calcium carbonate (15% *w*/*w*)	Barium sulphate (10% *w*/*w*)
Antibiotic	Gentamicin (2.5% *w*/*w)*	Gentamicin (2.5% *w*/*w*)
Condition for application	Preformed	Preformed
Type	Mobile articulating spacer	Mobile articulating spacer

**Table 2:** Surface roughness values of the two investigated spacer cements.

Spacer cement	Condyle		Tibia	
	Ra (in μm)	Rz (in μm)	Ra (in μm)	Rz (in μm)
CC–cement	1.166 ± 0.359	6.176 ± 1.613	1.560 ± 0.463	7.998 ± 2.219
BS–cement	1.127 ± 0.074	9.856 ± 0.457	1.086 ± 0.174	9.361 ± 1.523

**Table 3:** Characteristics of the two investigated spacer cements.

Particle parameters	CC-Cement	BS-Cement	*p*
Estimated total number of released particles	7.07 × 10^11^ ± 1.33 × 10^11^	10.38 × 10^11^ ± 1.17 × 10^11^	0.136
Equivalent circle diameter (ECD) in μm	0.378 ± 0.009	0.427 ± 0.010	0.020
Aspect ratio (AR)	1.730 ± 0.017	1.655 ± 0.0288	0.087
Roundness (R)	0.592 ± 0.005	0.592 ± 0.005	0.962
Form factor (FF)	0.674 ± 0.003	0.677 ± 0.005	0.574

**Table 4:** Wear behavior obtained on cement in the current study spacers in comparison to published studies on conventional polyethylene in total knee replacements. Values are presented as means ± standard deviation.

Study	Gravimetric Wear in mg per Million Cycles	Estimated Total Number of Particles Released per Million Cycles
Current study	*CC-cement:* 272.63 (±75.17) ^1^ *BS-cement:* 751.06 (±322.04) ^1^	*CC-cement:* 14.14 × 10^11^ (±4.62 × 10^11^) ^1^ *BS-cement:* 20.75 × 10^11^ (±4.06 × 10^11^) ^1^
Roy et al. [19]	0.06 (±0.06)	-
Reinders et al. [20]	8.0 (±0.9)	7.1 × 10^11^ (±1.0 × 10^11^)
Reinders et al. [21]	9.7 (±1.2)	3.93 × 10^11 1^
Kretzer et al. [22]	7.28 (±0.27)	-
Kretzer et al. [23]	10.55–16.08	2.63 × 10^11^–3.36 × 10^11^
Grupp et al. [24]	6.6 (±2.0)–9.7 (±1.3)	-

^1^ Converted to one million loading cycles for comparison.

## Abbreviations

The following abbreviations are used in the manuscript:

AMTI	Advanced Mechanical Technology Inc. (Watertown, MA)
ASTM	American Society for Testing and Materials
BS	Barium Sulphate
CC	Calcium Carbonate
EDX	Energy Dispersive X-ray analysis
ISO	International Standard Organization
PMMA	Polymethylmethacrylate
SEM	Scanning Electron Microscope
